# Oral and maxillofacial surgical services amid COVID-19 pandemic: perspective from Tanzania

**DOI:** 10.1186/s41182-020-00258-z

**Published:** 2020-08-17

**Authors:** Karpal Singh Sohal, Elison N. M. Simon, Boniphace Kalyanyama, Jeremiah Robert Moshy

**Affiliations:** 1 Department of Oral and Maxillofacial Surgery, Muhimbili University of Health and Allied Sciences, P.O. Box 65001, Dar es Salaam, Tanzania; 2 Department of Immunology and Microbiology, Muhimbili University of Health and Allied Sciences, Dar es Salaam, Tanzania

**Keywords:** COVID-19, Oral and maxillofacial surgery, Tanzania

## Abstract

The coronavirus disease 2019 (COVID-19) caused by the severe acute respiratory syndrome coronavirus 2 is a global pandemic that affects individuals from all walks of life. Considering that the virus can be passed on directly from person to person through respiratory droplets, contact, fomites, and saliva, the oral and maxillofacial surgeons are exposed to COVID-19 in their daily clinical duties. This is because of the nature of their work, which entails working within a short distance from patients’ oral cavity and upper airway. As such, there is a need for having locally tailored standard guidelines for managing patients with oral and maxillofacial conditions during the COVID 19 pandemic in Tanzania.

To the Editor,

The coronavirus disease 2019 (COVID-19) caused by the severe acute respiratory syndrome coronavirus 2 (SARS-CoV-2) was declared a global pandemic by the World Health Organization (WHO) on 11 March 2020 [1]. As of 5 May 2020, about 212 countries around the world had reported the presence of the disease [2]. In Tanzania, the first case was confirmed on 16 March 2020 [3]. COVID-19 affects individuals from all walks of life regardless of their social-economic status, and thus patients with various conditions affecting the oral and maxillofacial region are not exceptional. Due to the presence of a vast array of anatomical structures, the complex oral and maxillofacial region is affected by varied conditions ranging from developmental defects, infections, injuries, and pathologies [4]. The management of most of these conditions eventually requires a surgical approach, which must be carried out by an oral and maxillofacial surgeon (OMS).

The SARS-CoV-2 can be passed on directly from person to person through respiratory droplets, contact, fomites [5], and possibly saliva [6, 7]. Considering this, it is beyond doubt that the OMS is among the healthcare workers who are highly vulnerable to COVID-19 due to the area of work and the type of procedures done on daily basis [1]. The high-risk OMS face is attributed to the short distance between patients’ oral cavity and upper airway to the surgeons’ mouth/nose, and long duration of exposure or contact with the patient during surgical procedures. Further, the situation is compounded by constant contact with the patient’s secretions (saliva, mucus, blood, etc.) during different phases of diagnosis and disease management process [1]. This is worsened by the fact that they risk managing asymptomatic SARS-CoV-2 carriers who have the potential to transmit the disease considering that the incubation period of the virus is long and unpredictable (0–27 days, mean 6.4 days) [8].

In Tanzania, the current population is estimated to be 56 million [9], yet there are less than 30 practicing OMS in the whole country. Of these, about half, are at the Muhimbili National Hospital, located in the city of Dar es Salaam, which has a well-established oral and maxillofacial unit. This receives patients with different oral and maxillofacial conditions from all over the country. Normally, the surgical procedures that are performed in our center range from surgical extractions to excision of rather large tumors and management of trauma cases. These are carried out under local anesthesia, conscious sedation, and general anesthesia. On average about 70 patients are treated in the department of oral and maxillofacial surgery per day. Taking all this into consideration, and the fact that asymptomatic SARS-CoV-2 carriers have the potential to transmit the disease [8], the more patients the OMS comes into contact, the higher are the chances of contacting COVID-19.

There are several guidelines in managing patients during this pandemic era. Some institutions have laid out guidelines/recommendations on the management of patients with oral and maxillofacial conditions amid the COVID-19 pandemic [8, 10, 11]. Some of these are easily applicable in our setting while others may not be possible. The basic infection control practice such as hand hygiene practices, barrier techniques, the use of personal protective equipment (PPE) (face shield/goggles and surgical masks), disinfection of surfaces, and sterilization of equipment is very applicable and is routinely practiced in our setting.

In most developed nations, it has been proposed to postpone/scheduled elective procedures during this pandemic to protect the community, patients, and staff, while providing emergency care only [8, 12]. This, however, may not be very realistic in most developing countries including Tanzania, where patients seek treatment at late stages with advanced disease conditions. Our patients seek oral and maxillofacial care at an early stage only when they have unbearable pain or sustained traumatic injuries to the orofacial region. Cost implications are among the main causes of delay in reporting to a health facility in addition to the existing strong belief in traditional medicine among a considerable proportion of the population [13]. As such, the majority of the patients who are seen daily require emergency care like extraction of grossly carious teeth, incision and drainage of fascial space abscesses, and biopsies of advanced-stage malignant lesions.

Encouraging patients to seek online and telephonic consultations and fill in questionnaires before treatment may not be feasible in our setting. Though, the proposal that the patient’s body temperature should be measured using a non-contact infrared thermometer before treatment is possible. The reliability of body temperature, however, at times comes into question, since in cases where the patient presents with malignant disease (e.g., lymphoma) or fascial space infection, the body temperature may also rise. A thorough but quick history is taken from the patient concerning his/her condition and additional questions like travel history to COVID-19-affected locations, the presence of symptoms such as fever, shortness of breath, cough, and loss of smell/taste are also inquired.

In this institution, we have prioritized the surgical interventions to managing maxillofacial trauma, fascial space infections (e.g., Ludwig’s angina), malignant lesions (e.g., sarcomas and carcinomas), and any other condition in which delay could be detrimental to the patient’s life. For the cases of maxillofacial trauma, where applicable, closed reduction methods (e.g., maxillomandibular fixation) are preferred to open reduction and internal fixation. In a few selected cases with predictable favorable outcomes, some onco-surgical procedures are sometimes carried out under local anesthesia or conscious sedation. Performing SARS-CoV-2 tests to all patients admitted in oral and maxillofacial surgical wards on a routine basis as suggested by some authors [11] is not practical in our situation. Literature shows that conventional chest radiographs have the potential of giving a clue of the presence of COVID-19 [14]. Therefore, routine investigation such as a chest X-ray which is done in all cases in our setting may be relied upon as a preliminary indicator.

As the COVID-19 situation progresses, one may argue that closing oral and maxillofacial practices during the pandemic could reduce the chances of practicing OMS getting infected. However, in so doing it will increase the suffering of patients who require urgent oral and maxillofacial surgery care. These patients will in turn be directed to the emergency departments where the medical team is already overwhelmed with patients suffering from different conditions including COVID-19. It is our sole duty as OMS in Tanzania to protect both the patients and the medical team from any sort of infection and at the same time keep the healthcare system running efficiently.

In our center, in order to minimize the chances of contacting COVID-19, we have set local guidelines that should be followed. Patients are required to wash their hands with soap and running water and have their body temperature checked before entering the center. Inside the center, they are required to constantly wear a face mask and maintain a social distance of at least 6 feet apart. To all personnel involved in patient care, special sessions are organized for raising their awareness on COVID-19. The medical staff should take a thorough history from the patient and minimize the time spent with each patient without compromising standard treatment protocols. The decision of whether a given case is emergency or elective is made by a panel of not less than 3 specialists in the field of oral and maxillofacial surgery. Medical staff must wear protective gadgets (goggles, face shield, surgical masks, and aprons) whenever they attend patients. The face shields and goggles are disinfected while the mask and aprons are changed and disposed of appropriately after attending every patient. For all aerosol-generating procedures, powerful suction is used to remove blood and secretions from the site of operation. In the clinics, the operatories are disinfected every after attending patients, and the instruments are sterilized immediately after the procedures. Congestion of patients in the wards is minimized by shortening duration of hospital stay and, except when necessary, post-treatment follow-ups are scheduled with a long span of time.

In conclusion, it is plausible to continue with oral and maxillofacial services while maintaining strict preventing conditions against transmission of COVID-19. The creation of locally tailored standard guidelines for managing patients with the oral and maxillofacial conditions during the COVID-19 pandemic should be emphasized.

## Data Availability

The materials described in this letter are freely available from the corresponding author on reasonable request. Conclusions drawn in this letter reflect the authors’ views and not the institution of affiliation.

## Abbreviations

COVID-19: Coronavirus disease-2019
OMS: Oral and maxillofacial surgeon
PPE: Personal protective equipment
SARS-CoV-2: Severe acute respiratory syndrome coronavirus 2
WHO: World Health Organization
